# 3D Modeling of the Crystalline Lens Complex under Pseudoexfoliation

**DOI:** 10.3390/bioengineering9050212

**Published:** 2022-05-13

**Authors:** Leonor Jud, André P. G. Castro, Rui B. Ruben, Bernardo Feijóo, Filomena J. Ribeiro, Paulo R. Fernandes

**Affiliations:** 1 IDMEC, Instituto Superior Técnico, Universidade de Lisboa, 1049-001 Lisbon, Portugal; leonorjud@tecnico.ulisboa.pt (L.J.); paulo.rui.fernandes@tecnico.ulisboa.pt (P.R.F.); 2 ESTS, Instituto Politécnico de Setúbal, 2910-761 Setúbal, Portugal; 3 ESTG, CDRSP, Instituto Politécnico de Leiria, 2411-901 Leiria, Portugal; rui.ruben@ipleiria.pt; 4 Departamento de Oftalmologia, Hospital da Luz, 1500-650 Lisbon, Portugal; bfeijoo@hospitaldaluz.pt (B.F.); fjribeiro@hospitaldaluz.pt (F.J.R.); 5 Faculdade de Medicina, Universidade de Lisboa, 1649-028 Lisbon, Portugal

**Keywords:** pseudoexfoliation, lens complex, zonules, 3D modeling, biomechanics, finite element analysis

## Abstract

Pseudoexfoliation, one of the most frequent crystalline lens complex disorders, is prevalent in up to 30% of individuals older than 60 years old. This disease can lead to severe conditions, such as subluxation or dislocation of the lens, due to the weakening of the zonules. The goal for the present study was to understand the relevant biomechanical features that can lead to the worsening of an individual’s visual capacity under pseudoexfoliation. To this end, finite element models based on a 62-year-old lens complex were developed, composed by the capsular bag, cortex, nucleus, anterior, equatorial, and posterior zonular fibers. Healthy and pseudoexfoliative conditions were simulated, varying the location of the zonulopathy (inferior/superior) and the degenerated layer. The accommodative capacity of the models with inferior dialysis of the zonular fibers was, on average, 4.7% greater than for the cases with superior dialysis. If the three sets of zonules were disrupted, this discrepancy increased to 14.9%. The present work provides relevant data to be further analyzed in clinical scenarios, as these models (and their future extension to a wider age range) can help in identifying the most influential regions for the reduction of the visual capacity of the lens.

## 1. Introduction

Pseudoexfoliation affects up to 30% of people older than 60-year-old worldwide [1,2]. Under these circumstances, 70 million individuals are estimated to live with this condition, which decreases the accommodative capacity of the patients [3,4]. This age-related disease is often correlated with cataract and glaucoma conditions [5,6,7], so its early identification would be a plus for the prevention of such diseases. Other risks associated with this disease are the conditions of phacodonesis and, during surgery performed on the lens complex, multiplied zonular dialysis and vitreous loss.

Pseudoexfoliation is detected by the presence of the white pseudoexfoliative material in the anterior surface of the lens capsule. This syndrome is characterized by the pathological accumulation of an abnormal fibrillar extracellular material in ocular tissues, such as the lens capsule and the ciliary processes. Retinal and choroidal vessel density may also be affected, this being observed in optical coherence tomography angiography examinations. The risk of conditions, such as spontaneous subluxation or dislocation of the lens, in pseudoexfoliation patients can be up to ten times higher than for the case of healthy eyes [1,2,8,9,10].

Thus, this condition heavily affects the human crystalline lens, by altering the transmission and convergence of the light received by the eye into the retina. When the shape of the human crystalline lens is changed, with the support of the surrounding structures, the variation between near and distant vision becomes possible [2,5,11], according to the accommodation mechanism presented by Helmholtz. As the lens is subjected to changes in its structure throughout the life of an individual, its ability to accommodate decreases significantly [8,9].

The healthy lens is composed by its cortical and nuclear regions and is contained by an elastic membrane, also known as the capsular bag. This structure is then connected to the main ocular architecture by the zonular fibers, i.e., the suspensory ligament of the lens, which originate on the ciliary muscle. The fibers are divided in three different sets: the anterior, equatorial, and posterior zonule. Zonulopathy starts when the zonules become weaker and eventually are disrupted, reducing their support to the lens [12,13,14,15].

In detail, the abovementioned fibrillar bundles proliferate in the ciliary processes and the pre-equatorial lens epithelium, which are locations of zonular fiber insertion. The observation of the proliferative region of the lens denotes that the pseudoexfoliation clumps are produced by the lens epithelial cells. The effects of pseudoexfoliation syndrome in the capsule’s morphology are not significant on either of its poles, which present the same thickness and elasticity as in control eyes. The same does not apply to the attachment regions of the suspensory ligament. The deposition of the pseudoexfoliative material in these locations promotes zonular dialysis and weakness: the fibers are not only locally dislocated from the lens capsule, but also from the ciliary muscle, leading to their rupture due to the outburst of degenerative material [1,8,16,17]. Findings related to the location of the zonular dialysis reported that the zonular dialysis can be present in the inferior and superior regions of the structures [18], and that deposition of the pseudoexfoliative material is performed in the anterior–posterior direction [16,19,20]. The zonular weakness was also characterized according to in clock-hours, with each clock-hour accounting for a 30-degree zonular section, thus reflecting the angular extension of zonulopathy. Different levels of zonular fiber extension were found, where the dialysis could be extended for three hours (90-degrees), six hours (180-degrees), and throughout the whole zonular structure (360-degrees) [7,21].The present work aims to characterize the healthy lens complex through a tri-dimensional (3D) finite element (FE) model, as well as to depict the effects of zonulopathy in the lenticular system under conditions of the pseudoexfoliation syndrome. FE simulation is employed to vary the location and configuration of the zonular dialysis in 3D. The biomechanical effects of this disease, translated on the accommodation capacity of the lens, can raise awareness concerning the severity of the zonular dialysis and the best way to approach it in clinical scenarios.

## 2. Materials and Methods

### 2.1. The Healthy Lens Complex

**Figure 1:**
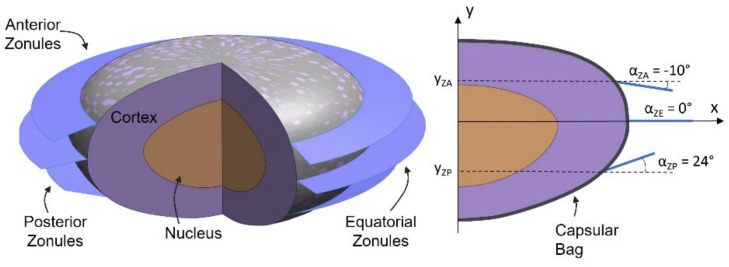
Geometry of the 62 year-old lens complex, sectioned 3D and axisymmetric views.

The 3D FE model for this study was based on a 62-year-old lens, given the significant prevalence of the pseudoexfoliation syndrome in the elderly population. The geometries of the nucleus and cortex of the lens were designed taking as a baseline the refractive index contours of the 62-year lens [22]. The resulting geometries are depicted in Figure 1, where the outer profiles of the nucleus and cortex were used to create axisymmetric structures [23], recurring to the axis of revolution ξ. These structures were modeled with 4-node linear tetrahedral elements, recurring to the ABAQUS^®^ (Dassault Systèmes Simulia Corp., Johnston, RI, USA). The capsular bag was designed as a membrane enveloping the cortex and, therefore, its geometry was constructed considering the outer profile defined for the cortex [23,24,25]. The capsule’s thickness was defined with a dimension of T_CB_ = 20 μm uniformly defined throughout its structure [26]. The capsule was modeled with 3-node triangular shell elements.

The zonular length of the three sets of zonules was l_Z_ = 1.5 mm, and the defining angles of the zonular sets were α_ZA_ = −10 deg, α_ZE_ = 0 deg, and α_ZP_ = 24 deg for the anterior, equatorial, and zonular fibers, respectively [26]. The three sets were defined with the same thickness value (T_Z_ = 10 μm) [27]. The three zonular sets were modeled with 3-node triangular membrane elements.

The lenticular components and the capsular bag were considered as having linear elastic isotropic material properties and being quasi-incompressible structures [25]. Accordingly, the Young’s modulus of the cortex and nucleus was E_C_ = 0.04 kPa and E_N_ = 0.82 kPa, respectively. The Poisson’s ratio was the same for both components (ν_C_ = ν_N_ = 0.47) [26]. The capsular bag had the following material parameters: E_CB_ = 1.5 MPa, ν_CB_ = 0.47 [26,28]. The zonular fibers were modeled as hyperelastic anisotropic structures, according to the constitutive properties based on the Holzapfel–Gasser–Ogden model [29]. The resulting material constants of the zonules that describe their constitutive behavior were C_10_ = 0.0583, D_1_ = 1.0286, k_1_ = 0.087 MPa, k_2_ = 21.75, and kappa = 0.3, inspired by the equivalent modeling of the annulus fibrosus of the intervertebral disc [30,31].

The boundary and interaction conditions imposed in this work related to the connection between structures, where the exterior surface of the nucleus was tied to the cortex interior facet, and the exterior surface of the latter structure was connected to the capsular bag’s interior membrane surface. A connection was also enforced between the interior edges of the zonules and the external insertion regions of the capsule. The most anterior and posterior poles of the lenticular components were only allowed movement in the optical axis direction, and the zonular movement was restricted in all directions except for the radial one. The disaccommodation process was simulated taking into account the displacement of the exterior edge of each of the zonular sets, δ_Z_ = 0.5 mm, that were stretched in the radial direction [12].

#### Zonular Geometry and Capsular Attachment

**Figure 2:**
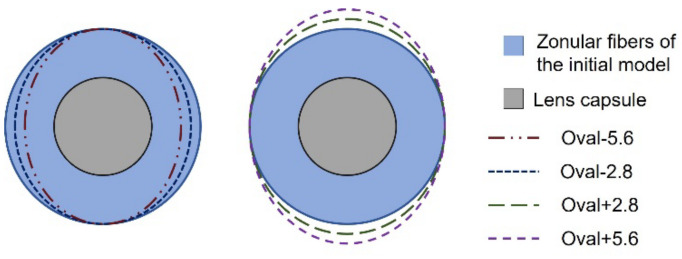
Frontal view schematic of the models with an oval zonular geometry.

The geometry of the zonules was then varied, given that these structures can have up to 5.6% difference between vertical and horizontal lengths, where the latter have a smaller dimension [32]. Accordingly, models were designed taking into account the representation depicted in Figure 2, and the measurements of the resulting zonular lengths are laid out in Table 1. It must be highlighted that the average fiber length (l_Z_) is increased. Additionally, the study of the boundary conditions related to the capsule and zonules was assessed. With the objective of avoiding the numerical irregularities originated by the zero-width rings [24] and understanding the impact of the insertion band width in the models, master surfaces were created in the capsule. These describe the belts of anchorage of the zonules and allow for the connection between the referred components.

The models developed are represented in Table 2, where models Band3 and Band7 have a band width correspondence with literature values [24,33], and the remaining models evaluate the intermediary values between the forementioned band widths. The gravitational force impact in the lens complex was assessed, where the density values of cortex, nucleus, and capsule were equivalent (δ_C_ = δ_N_ = δ_CB_ = 1099 kg/m^3^) [34], and the density of the zonules was determined as δ_Z_ = 1000 kg/m^3^ [35].

### 2.2. Pseudoexfoliation Syndrome

To implement the pseudoexfoliation model, zonular dialysis was simulated by reducing the thickness of the zonular set of fibers. This effect is due to the local dislocation of a portion of the fibers, whether they lift off from the ciliary muscle or from the lens capsule [8,16].

Two cases of zonular fiber separation were considered. A moderate situation, where 50% of the fibers were disconnected, leading to a resulting thickness of the afflicted areas of T_Z_ = 5 μm and a severe case, where the critically affected areas had 5% of healthy zonular thickness (T_Z_ = 0.5 μm) with transitional regions of moderate dialysis, where T_Z_ = 5 μm.

Taking into consideration the anterior–posterior direction of the zonulopathy conditions [16,18], the anterior zonules, the group of anterior and equatorial fibers, and the group of three sets of zonular fibers were degenerated. Nonetheless, for the sake of the individual characterization of each fiber set, the individual dialysis of the equatorial and posterior fibers was also modeled.

Given the fact that zonulopathy can either occur in the inferior or superior regions of the zonules [16,19], these two cases were simulated. Upon modeling the extension of zonular dialysis, the degeneration was accounted for one, three, six, nine, and 12 h, which corresponded to angles of 30, 90, 180, 270, and 360-degrees of zonulopathy.

**Figure 3:**
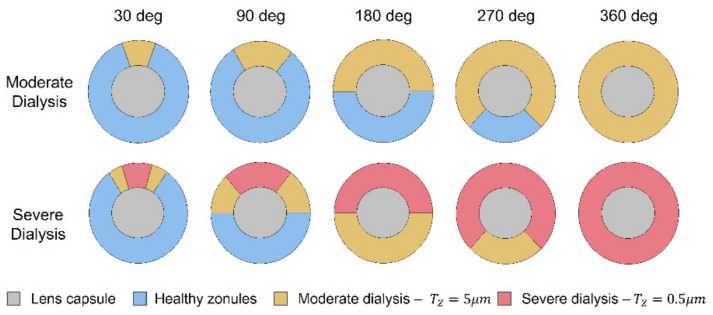
Frontal view schematic of the models with superior zonular dialysis.

Figure 3 portrays the schematic representation for the superior dialysis of the anterior zonules, for moderate and severe cases and for all angle cases of zonulopathy extension. Considering the progression of the pseudoexfoliation syndrome with regard to the previously mentioned factors, a total of 90 models were developed, to depict a gradual and progressive advancement of the disease in the lens complex.

The reported outputs include the lens thickness variation (∆T_L_), lens radius variation, (∆RL), total zonular force (F_Z_), the capsular bag strain, and stress ranges found for all the components [23].

Central optical power (*COP*), calculated as per Equation (1) [36], is the quantity that allows to link the clinical evaluation with the outputs of the FE simulation [24,36,37], as it provides the accommodative power of the lens, in diopters (D), for healthy and pathological conditions:

(1) $$COP=\frac{n_{l}-n_{p}}{r_{a}}+\frac{n_{l}-n_{p}}{r_{p}}-\frac{t\times {\left(n_{l}-n_{p}\right)}^{2}}{r_{a}\times r_{p}n_{l}}$$

where *n_l_* stands for the refractive index of the lens and *n_p_* the refractive index of the aqueous humour and vitreous body. Variable *t* represents the total thickness of the lens and *r_a_* and *r_p_* are the anterior and posterior radii of curvature, respectively. The variation of this quantity (∆*COP*) was calculated for all models, throughout the six simulation steps defined for the disaccommodation process [24,28]. This quantity was measured in the sagittal plane of the lens, where the radii of curvature were calculated assuming that the anterior and posterior surfaces of the lens were spherical.

## 3. Results

### 3.1. The Healthy Lens Complex

The lens under the initial conditions flattened ∆T_L_ = 14.20% and its radius increased ∆R_L_ = 7.87% during the disaccommodation process. The force acted on the zonular fibers was totaled as F_Z_ = 134.1 mN. The logarithmic strain endured by the capsular bag, LE_CB_, ranged from 0.0166 to 0.0825. The principal stress values found for the capsular bag in the validation model ranged from 41.53 kPa to 171.59 kPa, given that the regions under a greater amount of stress are the capsular bag’s anchorage rings, which connect to each of the zonular fiber sets. The stress in the nucleus and cortex was evaluated simultaneously, and values found for the principal stress on the structures ranged from −0.654 kPa to 0.841 kPa.

Conversely, the Von-Mises stress (VMS) ranged from 0.018 kPa to 0.210 kPa, and the stress distribution showed a greater stress magnitude in the regions proximal to the capsule’s attachment rings of the zonular fibers. This result shows a stress response of the cortex to the displacement of the fibers enforced in the disaccommodation process. The resulting amplitude of accommodation of the initial model was ∆*COP* = 1.57 D and the central optical power shows an increase in the first and second steps of the lens deformation, and a reduction throughout the rest of the disaccommodation process.

#### Zonular Geometry and Capsular Attachment

The evaluation of the geometry of the zonular fibers results revealed that the amplitude of accommodation of the lens showed a reduction throughout the evolution of the models presented in Table 1. For the model with the smallest average fiber length, Oval-5.6, the amplitude of accommodation had the highest value, ∆*COP* = 2.03 D. From then on, the amplitude of accommodation decreased, reaching the lowest value in model Oval + 5.6, where ∆*COP* = 1.38 D. The zonular force also denoted a reduction with the progression of the models in Table 1, reaching a minimum value of F_Z_ = 129.9 mN, for model Oval + 5.6. Given that these values were in a greater accordance with available literature [33,38], Oval + 5.6 depicted an optimized geometrical parameter model, then being selected to continue the process of optimization, regarding the capsular bag attachment parameter choice.

The evaluation of the interaction conditions, where the width of the capsular bag anchorage regions was varied, did not present relevant results, given that all the models from Band1 to Band7 showed similar behaviors, both in the stress and strain endured by the components of the lens complex and the accommodative capacity of the lens, as well as the force endured by the zonular fibers. Nonetheless, all these models showed a reduction in the accommodative amplitude and zonular force when compared with the model with zero-width rings of attachment in the capsule, given that there was a reduction of the endured effort in the capsule with the imposition of anchorage bands. As such, the selected model that portrayed optimized conditions was Band4, given its proximity with literature values [33,38].

### 3.2. Pseudoexfoliation Syndrome

The pseudoexfoliation syndrome led to the reduction of the capacity of the lens to change its geometry. Accordingly, the lens thickness variation reduced as the syndrome progressed. The evaluation of the individual impact of each of the zonules in the thickness variation of the lens showed that the equatorial zonules have the lowest impact in this quantity, both when moderately and severely dialyzed. The posterior and anterior zonules, when considered individually, lead to the same decrease of thickness variation of the lens (∆T_L_ = 8.55% for severe dialysis cases throughout the structure of the lens). Applying a severe degeneration in all zonular sets led to the most prominent discrepancy in lens thickness variation, given that for 360-degree dialysis this measurement was valued only ∆T_L_ = 4.28%.

When accounting for the lens radius variation results, contrarily to the analysis of the lens thickness variation, it was noted that the equatorial zonules play now an important role in the variation of the lens equator radius. This is due to the location of the equatorial fibers i.e., in the plane of the equator of the lens, where its radius is measured. The severe disruption of the equatorial fibers denotes a greater variation in the lens equator change in radius. When disrupted in the 12 hours, the lens radius variation presents a value of only ∆RL = 4.64%. As such, this set of fibers has a greater impact in the lens radius variation than the other two individual sets of zonules, the anterior and posterior zonules.

For the case of severe dialysis of the fibers, both the anterior and posterior zonules, when considered individually, show no effect in the radius variation of the lens. The moderate zonular disruption of all the considered zonular sets, does not have a great impact in the radius variation of the lens, even when applied to the complete structure of the zonules (360-degrees). For the three sets of zonules, when moderately afflicted throughout their complete structures, the lens equator shows a variation of ∆R_L_ = 6.42%, a reduction of 1.52% from the value found for the healthy model.

**Figure 4:**
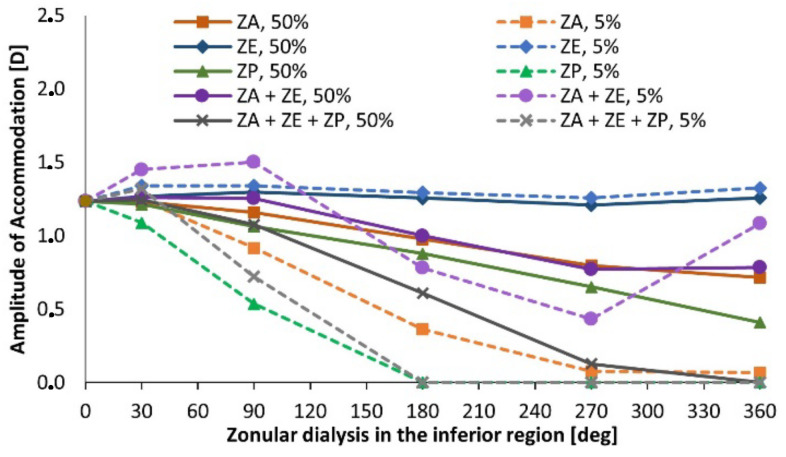
Amplitude of accommodation for pseudoexfoliative models with superior dialysis.

The accommodation amplitude results are depicted in Figure 4. The figure comprises the superior dialysis of the anterior zonules (ZA), equatorial zonules (ZE), and posterior ones (ZP), when degenerated in moderate (represented by the term 50%) and severe (5%) conditions. As for the case of no degeneration (healthy model), the model Band4 is portrayed with gravitational conditions, which were applied to all the pseudoexfoliation models. When evaluating the singular impact of the equatorial zonules in the accommodative capacity of the lens, one can recognize that both when moderately and severely dialyzed, the amplitude of accommodation attained by the lens shows no significant reduction for all cases of dialysis extent. Considering the singular performance of the dialyzed anterior fibers, when moderately afflicted, there is a reduction of the accommodation amplitude as the extent of dialysis increases, reaching a minimum value of ∆*COP* = 0.72 D for the fully afflicted anterior fibers, i.e., throughout their complete structure.

The moderate dialysis of the three zonular sets leads to the most acute reduction of accommodative amplitude in the models with 50% zonular thickness. The accommodation amplitude decreases significantly when increasing the dialysis degree, reaching a value of 0.08 diopters in the case with 270-degree dialysis, and 0 diopters when all zonular sets are completely disrupted.

The results found for the total force endured by the zonules during the deformation of the lens denote that all types of degeneration showed a downward slope in the resulting zonular force, as the extension of dialysis progressed from 30-degrees upwards. The zonular force due to the displacement of the fibers has a greater value in the exterior edge of the zonules, since this is the location of the boundary condition enforced on the fibers.

Due to the deteriorated conditions of the zonules, their stretching does not cause the same deformation on the capsule, which then translates to a reduction of the amount of stress that the capsule is under. Since the areas of greater stress in the capsule are the attachment regions to the anterior, equatorial, and posterior zonular fibers, as these components are thinned, the same displacement of the remaining fibers does not cause the same behavior in the capsule. The moderate disruption of each of the zonules led to a small decrease of the average stress endured by the capsule. The most extreme of the cases was for the moderate disruption of the anterior zonules, with VM_CB_ = 64.7 kPa, for 360-degree dialysis. The analysis of cases with severe zonulopathy led to the following deductions: the equatorial zonules are the ones with the lowest impact in this quantity, followed by the posterior zonules. The anterior zonules are the ones that, individually, cause the capsular bag to endure the smallest value of average Von-Mises stress, when dialyzed. The curve denoting a severe disruption of the anterior and equatorial fibers had a downward slope throughout the extension progression of dialysis, although more pronounced for the first 180-degrees of extension. From then on, the slope showed a more lenient inclination, and at 360-degrees dialysis, the average stress endured by the model with zonulopathy of anterior and equatorial fibers was VM_CB_ = 35.0 kPa.

**Figure 5:**
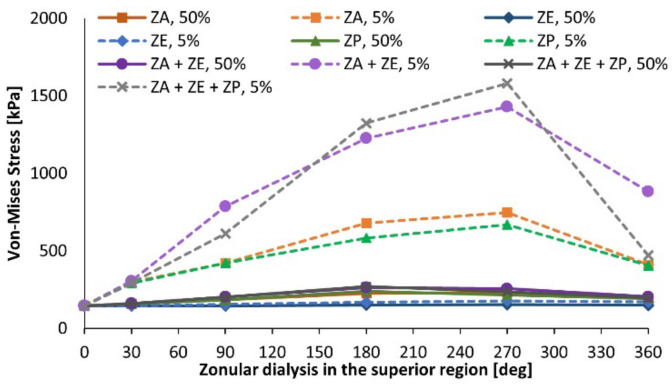
Maximum Von-Mises stress in the zonules for models with superior dialysis.

The maximum value of VMS that the zonules endure during disaccommodation of the lens is depicted in Figure 5. The models that accounted for severe dialysis of the zonules all showed a significant increase of the maximum zonular stress, apart from the equatorial zonules. Accordingly, the individual disruption of the anterior fibers showed a similar behavior to the one of the individual posterior fibers, where there was an increase of the maximum stress endured by the zonules up to 270-degree dialysis extension, followed by a reduction from the forementioned cases to the ones with 360-degree dialysis. The same results were found for the cases of dialysis of the groups of zonules (ZA + ZE and ZA + ZE + ZP), where an increase of stress was observed up to 270-degree dialysis extension, and a reduction observed for the dialysis of the whole structure. For the case of the severe disruption of the anterior and equatorial zonules, the maximum VMS for 270- and 360-degree zonulopathy extensions was 1429 kPa and 881 kPa, respectively. When examining the zonular areas that endured the greater amount of stress when under pseudoexfoliative conditions, the case of severe disruption of the anterior zonules was analyzed, for 90-degree extension.

The 90-degree region with severe disruption of the zonular fibers, where T_Z_ = 0.5 μm, is the one that endures the greater amount of stress in the area proximal to the capsular bag attachment. The areas of moderate disruption on both sides show a reduction of the stress endured, when compared with the severely damaged region. Lastly, the healthy region of zonules had the smallest amount of stress concentration. Nonetheless, the healthy region denotes a greater stress concentration in the areas closer to the moderately dialyzed regions. These results suggest that the stress endured by the diseased regions propagates to the healthy zonules, which then causes a progression of the degeneration, given that the latter fibers are now under a greater amount of stress that expected in healthy conditions.

There was an overall reduction of the stress endured by the cortex and nucleus, as the pseudoexfoliation syndrome evolved and reached more advanced stages. The analysis of the equatorial fiber disruption shows that although the moderate dialysis does not cause a significant decrease in the stress endured by the lens, the severe zonulopathy of the equatorial zonules has one of the most significant impacts in the stress reduction endured by these components. The anterior zonule disruption has an inverse effect in the cortical and nuclear stress since its dialysis leads to an increase of the resulting maximum stress.

Towards understanding the relevant disparities between the two groups of models (with inferior and superior dialysis), a percentual approximation error was computed between the values of superior and analogous inferior models, resorting to Equation (2):

(2) $$\lambda \left[\%\right]=\frac{{\mathrm{value}}_{\inf}-{\mathrm{value}}_{\sup}}{{\mathrm{value}}_{\sup}}\times 100$$

Note that the presented variable can have negative values: when the referred value for the inferior dialysis is smaller than the superior one, λ has a negative value, to highlight this behavior. The results found for the lens thickness variation, lens radius variation, and average capsular stress showed a negligible difference between models with superior and inferior origins.

The total zonular force had higher values for all the cases where there was a superior dialysis, i.e., the approximation error had negative values for all the comparisons between inferior and superior cases. The average approximation error was λ = 1.6%. These results show that when there is a degeneration with a superior origin, the zonular fibers endured a greater amount of force when achieving the same displacement as an analogous lens complex with inferior zonulopathy.

**Figure 6:**
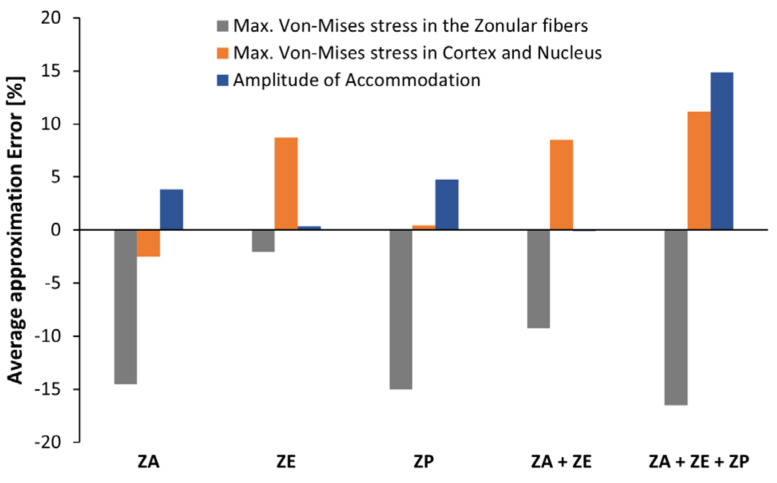
Comparison between superior and inferior values for maximum Von-Mises stress in the zonules, in the lens, and amplitude of accommodation.

The most important differences between the two types of degeneration were observed in the maximum VMS endured by the zonules, the maximum VMS endured by the lenticular components, and finally, the accommodation amplitude. These results are represented in Figure 6.

The average approximation error for each of the zonular groups considered denoted that the stress endured by models with superior dialysis origin endured a greater amount of stress than the analogous models with inferior dialysis origin, as defined by the negative values of λ. These results imply that the zonulopathy causes a greater amount of stress in the zonules when its origin has a superior location. Consequently, since the healthy zonular fibers suffer a greater disruption as the stress magnitude increases, the same fibers would be under a greater degeneration when in the presence of a superior dialysis, when compared with the analogous case of inferior dialysis.

The disparity between the superior and inferior values of the maximum VMS in the cortical and nuclear components of the lens is also depicted in Figure 6. The anterior zonular fiber disruption showed a peculiar behavior, when compared with the rest of the zonular sets: the average approximation error between superior and inferior values was valued as λ = 2.5%, denoting that the superior dialysis caused a greater stress in the cortex and nucleus than the inferior dialysis. However, all the other sets had an opposite behavior, where the average approximation error was positive, showing a greater stress in the models with inferior dialysis than models with a superior one.

The impact on the amplitude of accommodation was compared between inferior and superior dialysis for the different zonular fiber evaluations. The equatorial fibers, given that they have no significant impact in the accommodative capacity of the lens, as can be observed in Figure 4, did not present a significant difference between the inferior and superior dialysis values, given that their approximation error was λ = 0.4%.

The outcomes for the maximum VMS in the zonules and the accommodation amplitude of the lens seem to indicate that the superior dialysis leads to a more severe degeneration of the zonules and the visual capacity of the lens. The inferior dialysis leads to a less severe reduction of the accommodation amplitude of the lens and the inferior dialysis presents smaller stress magnitudes in the zonules, which then leads to a moderate progression of the zonulopathy condition.

## 4. Discussion

The main objective of this work was to address the challenge of determining the biomechanical and optical behavior of the human lens complex under healthy and diseased conditions. A novel 3D model for the healthy lens complex was built, where the biomechanical effects of both geometrical changes in the zonular fibers and the boundary conditions of the capsular bag were assessed. The variation of the geometrical parameters of the zonules, where the vertical length of the fibers was 5.6% greater than the horizontal one, led to results that are more relevant in terms of the accommodative capacity and biomechanical behavior of the lens. The assessment of the boundary conditions of the capsular bag, where the anchorage regions to the zonules were altered, led to the conclusion that when there is a zero-width insertion ring as the surface of attachment to the capsule, the values of force and accommodative amplitude are greater than for the cases where the capsule has bands of insertion with width greater than zero.

The outcomes related to the pseudoexfoliation syndrome seem to indicate that this disease has a significant impact on the accommodative capacity of the lens, exhibiting null accommodation amplitude for the most severe cases of zonular dialysis. The location of the zonulopathy in the lens complex had a noticeable impact in the progression of the disease and the accommodative capacity of the lens: the superior dialysis presents optimal conditions to further the dialysis of the fibers and reduce the accommodative amplitude of the lens, when compared with the inferior dialysis location. Accordingly, the accommodative capacity of the inferior models was, on average, 4.7% greater than for the cases with superior dialysis. When the three sets of zonules were disrupted, this discrepancy increased significantly, reaching an average value of 14.9% for the mentioned models, and with a maximum value of 60.1%.

This work led to the collection of original data concerning the healthy lens complex configuration, as well as the pseudoexfoliation syndrome and its effect in the studied system. It must be highlighted that literature data on pseudoexfoliative conditions are very limited in what concerns to modelling, hence the innovative nature of the present work. Future work shall not only involve other diseases of the human lens complex, such as the formation of cataract in the crystalline lens, but also models in different age ranges. Finally, the methodology applied towards the construction of the models in this dissertation could also be applied for cases in post-surgical perspectives, such as cataract surgery, where the nucleus and cortex are removed and substituted by an intraocular lens.

## Figures and Tables

**Table 1:** Models developed to assess the geometry of the zonular fibers.

Model	Vertical Length [mm]	Horizontal Length [mm]
Oval-5.6	1500	1416
Oval-2.8	1500	1458
Initial Model	1500	1500
Oval + 2.8	1542	1500
Oval + 5.6	1584	1500

**Table 2:** Band widths of the capsular anchorage regions for different model configurations.

Model	Anterior Band Width [mm]	Equatorial Band Width [mm]	Posterior Band Width [mm]
Band1	0.167	0.167	0.133
Band2	0.333	0.333	0.267
Band3	0.500	0.500	0.400
Band4	0.590	0.590	0.548
Band5	0.680	0.680	0.695
Band6	0.770	0.770	0.843
Band7	0.860	0.860	0.990

## Data Availability

The generated FE models can be made available upon reasonable request to the corresponding author.
